# Codon usage studies and epitope-based peptide vaccine prediction against *Tropheryma whipplei*

**DOI:** 10.1186/s43141-022-00324-5

**Published:** 2022-03-07

**Authors:** Amit Joshi, Sunil Krishnan, Vikas Kaushik

**Affiliations:** School of Bioengineering and Biosciences, Lovely Professional University, Phagwara, Punjab India

**Keywords:** *Tropheryma whipplei*, Synonymous codons, Ribosomal RNA, Gastroenteritis and codon usage

## Abstract

**Background:**

The *Tropheryma whipplei* causes acute gastroenteritis to neuronal damages in *Homo sapiens*. Genomics and codon adaptation studies would be helpful advancements of disease evolution prediction, prevention, and treatment of disease. The codon usage data and codon usage measurement tools were deployed to detect the rare, very rare codons, and also synonymous codons usage. The higher effective number of codon usage values indicates the low codon usage bias in *T. whipplei* and also in the 23S and 16S ribosomal RNA genes.

**Results:**

In *T. whipplei*, it was found to hold low codon biasness in genomic sets. The synonymous codons possess the base content in 3rd position that was calculated as A3S% (24.47 and 22.88), C3S% (20.99 and 22.88), T3S% (21.47 and 19.53), and G3S% (33.08 and 34.71) for 23s and 16s rRNA, respectively.

**Conclusion:**

Amino acids like valine, aspartate, leucine, and phenylalanine hold high codon usage frequency and also found to be present in epitopes KPSYLSALSAHLNDK and FKSFNYNVAIGVRQP that were screened from proteins excinuclease ABC subunit UvrC and 3-oxoacyl-ACP reductase FabG, respectively. This method opens novel ways to determine epitope-based peptide vaccines against different pathogenic organisms.

## Background

*Tropheryma whipplei* is an actinobacteria pathogen causing Whipple’s disease in *Homo sapiens*. This pathogenic problem was discovered and found to be associated with gastroenteritis, endocarditis, and neuronal damages in Caucasian individuals [1]. Regardless of this, its lethal impact was additionally seen in canines [2]. The credit for its name and disclosure was connected with honorable Nobel laureate G. H Whipple, who performed many explorations for lipodystrophy (malfunctioned lipid biosynthesis and ingestion) brought about by *T. whipplei* [3] has a broad-spectrum infection. Caucasian populaces, kids, sewage, and farming specialists were discovered to be generally influenced by this illness. The bacterium causes immunomodulation with an extended IL-16 discharge, IL-10 synthesis, and dysregulation of mucosal T-helper cells. Further immunological irregularities were depicted because of Whipple’s disease’s multifaceted nature [4]. Clinical side effects of this infection were seen as extreme looseness of the bowels, loss of body weight, and weakness among patients [5]. *T. whipplei* assaults lamina propria of the gastrointestinal tract and targets macrophages for its replication [6]. Sequencing of two strains of *T. whipplei* (Twist and TW 08/27) was effectively led by the French researchers that already open scope for genomic examination and improvement of better treatment procedures for this lethal sickness; in their investigation, it was discovered that this actinobacterium has low GC content (46%) in correlations with other relatives of a similar order [7].

Current medicines like doxycycline, hydroxychloroquine, and trimethoprim/sulfamethoxazole must be used for almost 2 years and lifetime follow-up for patients [8, 9]. Later in silico concentrates on epitope-based vaccine design can become conceivable prophylaxis for Whipple’s illness [10]. This actinobacterium has a huge encoding of surface proteins, while some are additionally connected with the enormous substance of noncoding redundant DNA. This genome additionally shows the fluctuation in genomic sets, including phase variations causing the modifications of cell proteins; this shows the importance of immune bypass and association with the host genome [1, 7]. Such uncommon genomic trademark highlights of bacterium open wide scope in discovering codon utilization patterns to uncover characteristic and mutational determination. Codons contained 3 nucleotides in sequence and coded for a particular amino acid or as a STOP codon for translation. The differences in codon usage are differences defined in codon usage bias. Equivalent codon utilization in numerous prokaryotic unicellular life forms is consistently connected with the directional mutational inclination and translational choice [11]. Other elements like replication-translation determination, protein hydropathy, can likewise have a critical impact [12]. In some microbial pathogen species, mutational predisposition was discovered to be strand explicit, and those living beings show differed interchangeable and nonequivalent codon utilization [13]. This examination not just give experiences about characteristic and mutational determination pressures acting at genomic levels of *T. whipplei* yet besides offer a superior cognizance of transformative improvements in this host-versatile bacterium. This computational examination uncovered the data concerning profoundly translated proteins and enzymes of this bacterium, and the conceivable amino acids that can be considered in epitope-based prophylaxis plan to get the inhibitory effect on bacterial action on its host or to create a better conceivable treatment like in immunoinformatics-based recent studies [14, 15]. Ribosomal RNA (16S and 23S) codon usage patterns were analyzed here to determine the changes associated with evolutionary or phylogenetic patterns of the bacterium. In this study, we also revealed epitope-based peptide vaccine candidates against *Tropheryma whipplei*. The aim of the study is to determine codon usage patterns in *T. whipplei*, and on the basis of that we predicted epitope-based vaccine candidate by deploying latest bioinformatics tools.

## Methods

### Codon data retrieval

To measure the codon usage bias, retrieved codon usage tables from codon and codon pair usage tables (CoCoPUTs) database. This database showed the relative frequency that different codons are used in genes in *T. whipplei* RefSeq data. Similarly, codon-pair usage tables displayed the counts of each codon pair in the CDSs of *T. whipplei* genomic data (RefSeq) and calculated codon-pair usage bias.

### Retrieval of genomic data and codon usage table

The complete nucleotide sequences of *T. whipplei* strains. The selected FASTA sequences of Twist 16S ribosomal RNA and 23S ribosomal RNA were retrieved from the NCBI Refseq database (https://www.ncbi.nlm.nih.gov/nuccore). The codon usage dataset was retrieved from the Codon Usage Database (http://www.kazusa.or.jp/codon/).

### Genomic sequence optimization

All codons in the original sequence of *T. whipplei* strains are replaced with the corresponding redundant codon having the highest codon usage frequency. ATGme tool [16] was used to identify rare codons and accordingly optimize genomic sequences (http://www.atgme.org/). Genomic sequences in FASTA format pasted in the search box, and codon usage table pasted in the respective interface and processed the data for analysis of rare codons and sequence optimization.

### Codon usage measurements

From the identified genomic sequences of ribosomal RNA, nucleotide composition was computed. The G + C composition of 1st, 2nd, and 3rd positions and GC1s, GC2s, and GC3s in the codons were discovered for the frequency and mean frequency identification. The frequency of synonymous third position codon and percentage, i.e., A3, T3, G3, and C3 and %A3s, %C3s, %T3s, and %G3s, respectively, was calculated. To measure the bias of synonymous codons, the effective number of codons (E*N*C) was identified. Additionally, codon usage, codon usage per thousand, and relative synonymous codon usage (RSCU) were also calculated using “CAIcal” tool availed from https://ppuigbo.me/programs/CAIcal/.

### Epitope-based vaccine prediction

Proteomic data for *Tropheryma whipplei* was accessed from NCBI GenBank database, and then allergenicity was estimated by deploying AllergenFP server [17]. NetMHCIIpan-4.0 server [18] was used to screen epitopes from selected proteins that can interact with human leukocyte antigen (HLA) proteins. VaxiJen 2.0 tool [19] was used to reveal antigenicity of screened epitopes. Epitopes structure was predicted by using PEP-FOLD 3.5 [20], and HLA allelic determinant HLA DRB1_0101 (PDB-ID:1AQD) was retrieved from RCSB-PDB database. Biochemical properties for epitopes were calculated by using ProtParam tool of ExPASy web server.

Molecular docking between epitopes and HLA determinants was done by using PatchDock [21], FireDock, and DINC web tool [22]. These tools not only assist in docking in user-friendly approach but also calculate different parameters like global energy, atomic contact energy, and binding energy for docked complexes.

## Results

### Identified codons and calculated usage bias

**Fig. 1:**
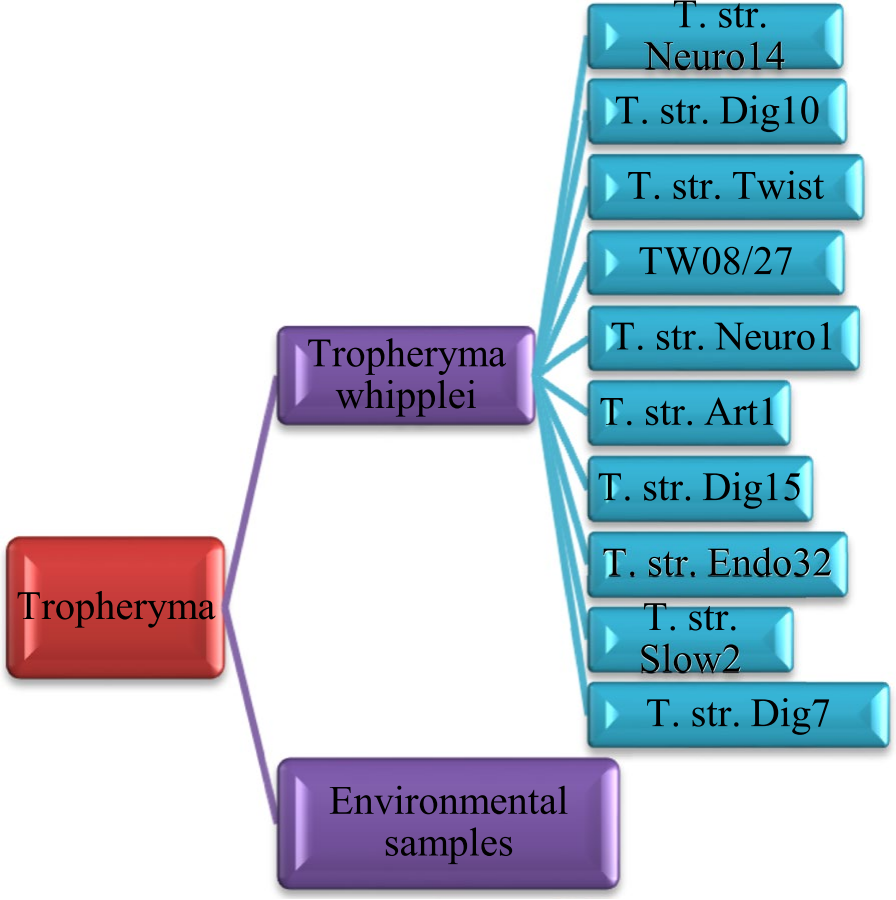
Taxonomy and strains of *Tropheryma whipplei*

**Fig. 2:**
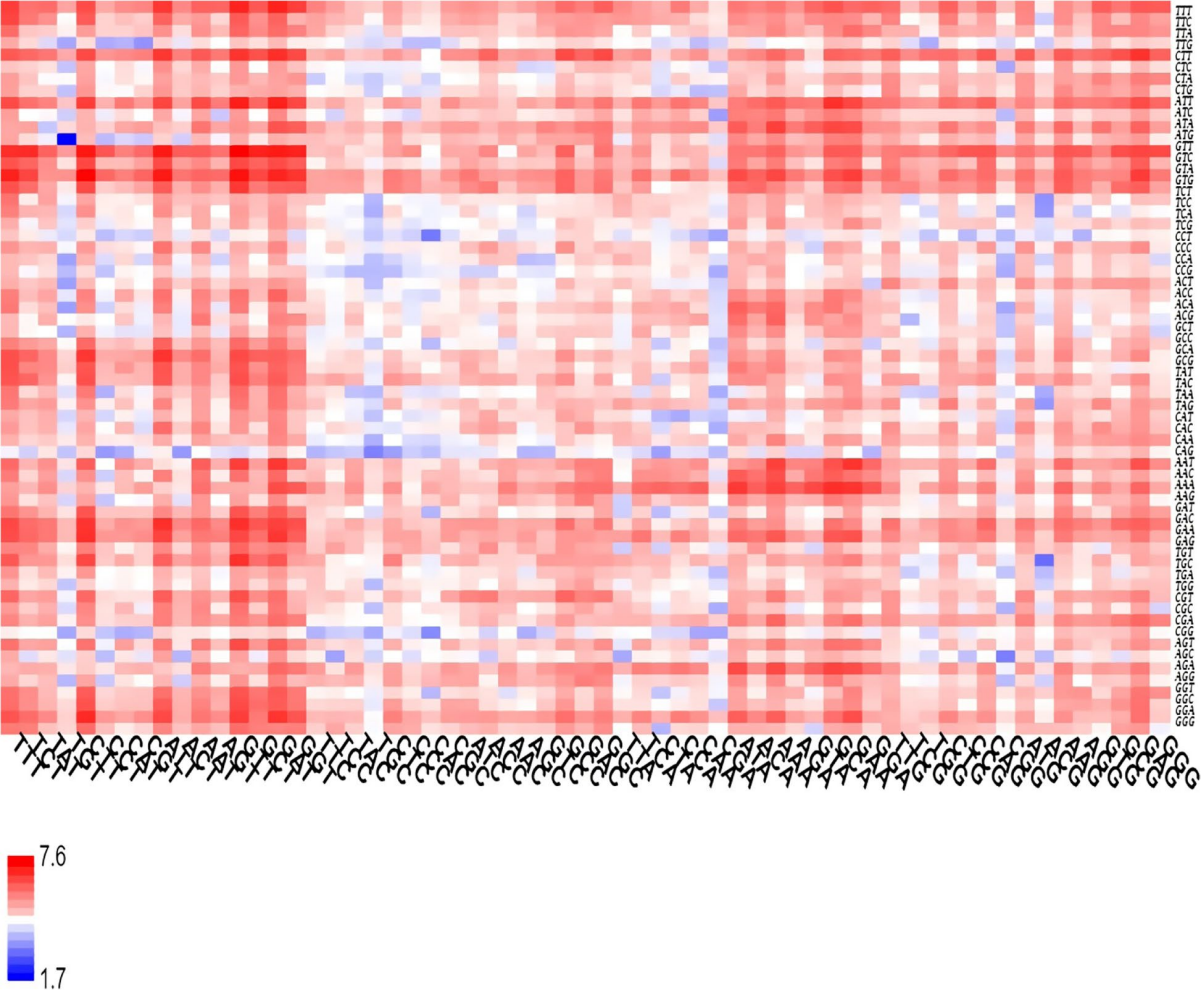
Heatmap of log-transformed codon usage

The codon-pair usage table and dinucleotide usage data were identified from the CoCoPUTs database [23, 24]. The *T. whipplei* taxonomy ID or taxid (2039) was verified by NCBI’s taxonomy tool, and the taxonomy was illustrated in Fig. 1. The log-transformed codon-pair frequency heat map was discovered from the data analysis as illustrated in Fig. 2. The degree of ENC values ranges from 20 to 61 [25]. If the value is 20, then one codon coding for each amino acid and value ranged to 61 means all the synonymous codon was used for each amino acid. The ENC value computed in our analysis was 56.138, which means more than one codon was used for each amino acid. The ENC value should be ≤ 35 for significant codon bias [26]. So, the higher ENC value indicates the low codon usage bias in *T. whipplei*. The ENC value details are demonstrated in Table 1.

**Table 1:** Effective number of codon pairs for each *T. whippelli*

ENc	ENcp	ENc (GC corrected)	ENcp (GC corrected)	Genetic code
56.138	54.026	57.212	54.910	Standard code

**Fig. 3:**
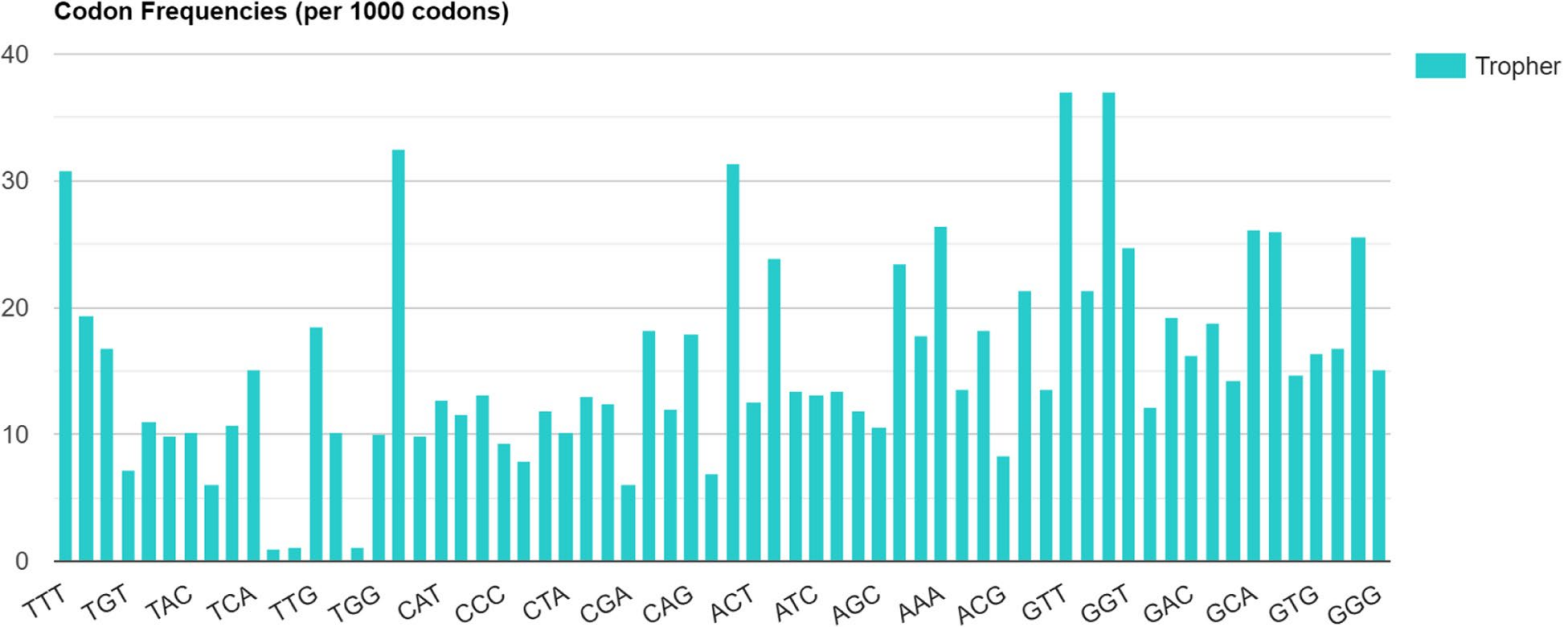
Codon frequencies of *Tropheryma whipplei*

**Fig. 4:**
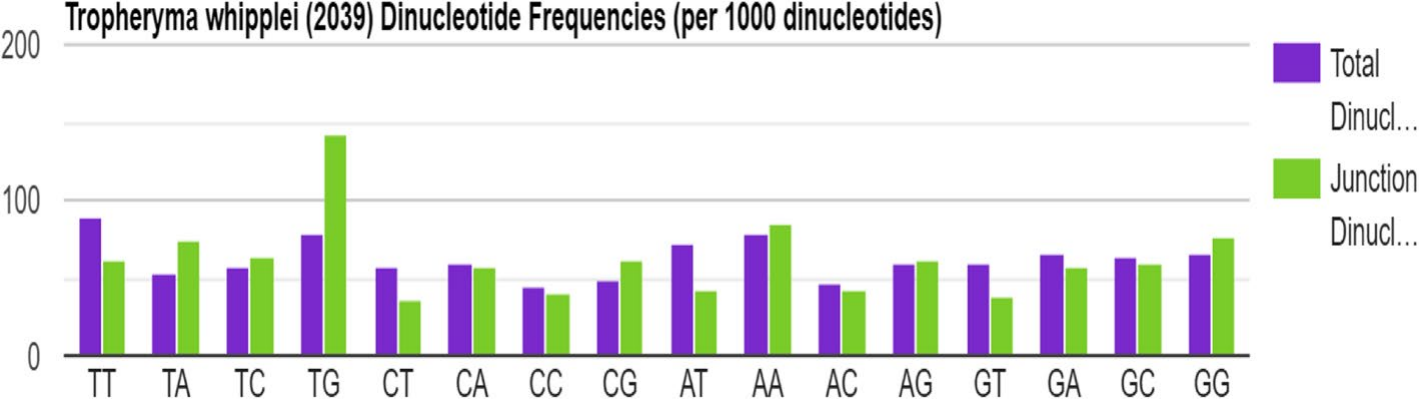
Dinucleotide frequencies of *Tropheryma whipplei*

The codon usage details are summarized in the Table 2, and the codon usage frequency per 1000 codons is illustrated in Fig. 3. The RefSeq (*n* = 859) of T*. whipplei* had 88597 CDSs and 28006357 codons. Table 2 illustrated the CDS and its codon pair. The codons GTT (37.06), GAT (37.03), CTT (32.53), and TTT (30.88) were identified as the highest usage frequency (frequency value shown in bracket). Dinucleotide frequencies per 1000 dinucleotide are demonstrated in Fig. 4.

**Table 2:** *Tropheryma whipplei* RefSeq codon table contains 88597 CDSs (28006357 codons)

Codon	Usage frequency	No. of codons	Codon	Usage frequency	No. of codons	Codon	Usage frequency	No. of codons	Codon	Usage frequency	No. of codons
**TTT**	30.88	(864933)	**TCT**	19.41	(543618)	**TAT**	16.82	(470999)	**TGT**	7.23	(202580)
**TTC**	11.07	(309980)	**TCC**	9.94	(278337)	**TAC**	10.24	(286912)	**TGC**	6.09	(170524)
**TTA**	10.79	(302319)	**TCA**	15.06	(421837)	**TAA**	1.00	(27960)	**TGA**	1.11	(31013)
**TTG**	18.49	(517778)	**TCG**	10.24	(286768)	**TAG**	1.14	(31981)	**TGG**	10.09	(282675)
**CTT**	32.53	(910922)	**CCT**	9.92	(277793)	**CAT**	12.79	(358300)	**CGT**	11.66	(326630)
**CTC**	13.10	(366773)	**CCC**	9.27	(259711)	**CAC**	7.92	(221772)	**CGC**	11.85	(331835)
**CTA**	10.11	(283081)	**CCA**	13.02	(364568)	**CAA**	12.40	(347268)	**CGA**	6.07	(169892)
**CTG**	18.23	(510468)	**CCG**	12.02	(336672)	**CAG**	17.96	(503036)	**CGG**	6.98	(195455)
**ATT**	31.40	(879451)	**ACT**	12.53	(350913)	**AAT**	23.82	(667060)	**AGT**	13.41	(375656)
**ATC**	13.10	(366940)	**ACC**	13.40	(375396)	**AAC**	11.87	(332502)	**AGC**	10.61	(297274)
**ATA**	23.41	(655721)	**ACA**	17.74	(496749)	**AAA**	26.46	(741084)	**AGA**	13.59	(380649)
**ATG**	18.25	(511118)	**ACG**	8.30	(232504)	**AAG**	21.39	(598923)	**AGG**	13.55	(379598)
**GTT**	37.06	(1037894)	**GCT**	21.35	(597805)	**GAT**	37.03	(1037065)	**GGT**	24.77	(693723)
**GTC**	12.15	(340402)	**GCC**	19.26	(539448)	**GAC**	16.30	(456523)	**GGC**	18.74	(524935)
**GTA**	14.34	(401675)	**GCA**	26.13	(731733)	**GAA**	25.97	(727374)	**GGA**	14.66	(410657)
**GTG**	16.39	(459063)	**GCG**	16.86	(472115)	**GAG**	25.54	(715420)	**GGG**	15.16	(424597)

### *Tropheryma whipplei str. Twist* codon usage table

*Tropheryma whipplei* strain Twist complete sequence of 23S and 16S ribosomal RNA genes were composed of 3102 base pairs and 1521 base pairs, respectively. *Tropheryma whipplei* Twist strain’s CDS, codons, frequency per thousand, and the number of codons details are summarized in Tables 3 and 4. These codon usage tables were used for the identification of rare codons and sequence optimization.

**Table 3:** ***Tropheryma whipplei*** str. Twist **808 CDS’ (266294 codons)** codons, frequency per **thousand, and in bracket number of codons**

Codon	Frequency (no. of codon)	Codon	Frequency (no. of codon)	Codon	Frequency (no. of codon)	Codon	Frequency (no. of codon)
UUU	30.5 (8121)	UCU	19.7 (5246)	UAU	17.2 (4590)	UGU	7.3 (1938)
UUC	11.5 (3066)	UCC	10.1 (2690)	UAC	10.5 (2790)	UGC	6.1 (1626)
UUA	10.9 (2906)	UCA	15.4 (4100)	UAA	0.9 (250)	UGA	1.1 (281)
UUG	18.4 (4894)	UCG	9.9 (2643)	UAG	1.0 (277)	UGG	10.2 (2710)
CUU	31.8 (8461)	CCU	10.6 (2826)	CAU	12.8 (3409)	CGU	11.6 (3079)
CUC	13.1 (3492)	CCC	9.8 (2620)	CAC	7.9 (2111)	CGC	11.6 (3085)
CUA	10.6 (2832)	CCA	13.5 (3588)	CAA	12.5 (3316)	CGA	6.0 (1585)
CUG	18.3 (4871)	CCG	11.6 (3095)	CAG	18.4 (4889)	CGG	6.9 (1832)
AUU	30.6 (8157)	ACU	12.7 (3392)	AAU	23.7 (6313)	AGU	13.1 (3497)
AUC	13.2 (3503)	ACC	14.2 (3776)	AAC	11.9 (3179)	AGC	10.7 (2855)
AUA	23.3 (6209)	ACA	19.6 (5223)	AAA	26.2 (6970)	AGA	13.6 (3613)
AUG	18.0 (4784)	ACG	8.2 (2176)	AAG	21.2 (5642)	AGG	13.2 (3516)
GUU	36.7 (9774)	GCU	21.3 (5660)	GAU	36.3 (9679)	GGU	24.9 (6640)
GUC	12.2 (3247)	GCC	19.4 (5172)	GAC	16.1 (4283)	GGC	18.8 (5007)
GUA	14.7 (3916)	GCA	26.1 (6939)	GAA	25.2 (6702)	GGA	14.8 (3952)
GUG	16.6 (4431)	GCG	16.3 (4340)	GAG	24.7 (6586)	GGG	14.8 (3942)
**GC percent information**				**Coding GC 46.46%**	**Ist letter GC 54.59%**	**2nd letter GC 42.30%**	**3rd letter GC 42.48%**

**Table 4:** ***Tropheryma whipplei*** TW08/27**783 CDSs and 261028 codons**, frequency **per thousand, and in bracket number of codons**

Codon	Frequency (no. of codon)	Codon	Frequency (no. of codon)	Codon	Frequency (no. of codon)	Codon	Frequency (no. of codon)
UUU	30.4 (7947)	UCU	19.8 (5158)	UAU	17.4 (4531)	UGU	6.9 (1813)
UUC	11.4 (2984)	UCC	10.3 (2683)	UAC	10.5 (2743)	UGC	5.7 (1496)
UUA	10.7 (2802)	UCA	15.6 (4063)	UAA	1.0 (251)	UGA	1.0 (265)
UUG	17.7 (4611)	UCG	9.8 (2567)	UAG	1.0 (267)	UGG	10.0 (2603)
CUU	31.9 (8314)	CCU	10.6 (2762)	CAU	12.8 (3343)	CGU	11.5 (2996)
CUC	13.4 (3509)	CCC	9.8 (2560)	CAC	7.8 (2034)	CGC	11.5 (3008)
CUA	10.8 (2829)	CCA	13.8 (3610)	CAA	12.6 (3276)	CGA	5.8 (1513)
CUG	18.2 (4741)	CCG	11.5 (3014)	CAG	18.4 (4793)	CGG	6.7 (1747)
AUU	30.7 (8013)	ACU	12.8 (3352)	AAU	23.7 (6193)	AGU	13.1 (3413)
AUC	12.9 (3377)	ACC	14.6 (3803)	AAC	12.1 (3149)	AGC	10.7 (2781)
AUA	23.6 (6166)	ACA	20.1 (5243)	AAA	26.2 (6829)	AGA	13.6 (3546)
AUG	17.9 (4662)	ACG	8.1 (2108)	AAG	21.2 (5533)	AGG	13.1 (3409)
GUU	36.9 (9638)	GCU	21.3 (5567)	GAU	36.7 (9577)	GGU	25.0 (6521)
GUC	12.2 (3193)	GCC	19.6 (5111)	GAC	16.2 (4218)	GGC	18.7 (4884)
GUA	14.7 (3835)	GCA	26.1 (6821)	GAA	25.2 (6578)	GGA	14.9 (3879)
GUG	16.3 (4256)	GCG	16.2 (4239)	GAG	24.9 (6488)	GGG	14.6 (3813)
**GC percent information**				**Coding GC 46.41%**	**Ist letter GC 54.66%**	**2nd letter GC 42.27%**	**3rd letter GC 42.29%**

### Rare and very rare codons

The analysis resulted from usage data, original sequence, and optimized sequence. *Tropheryma whipplei* strain Twist 23S ribosomal RNA gene sequence analyzed usage data predicted GTT and GAT (36.7% and 36.3 %) had the high frequency in codon usage. TAA, TAG, and TGA code as “STOP” had the lowest usage frequency percentage ((0.9 %, 1.0 % and 1.1 %) and found these are the very rare codons. The rare codons are CGA, TGC, CGG, TGT, CAC, ACG, CCC, and TCG. The stop codons are terminating the protein translation process [27]. The details of rare codons and very rare codons (code as, count, and percentage of usage frequency) of 23s and 16S rRNA were summarized in Tables 5 and 6.

**Table 5:** *Tropheryma whipplei* strain Twist 23S ribosomal RNA gene

Codon	Codes as	Usage frequency ‰	Count
**TAA**	STOP	0.9	14
**TAG**	STOP	1	26
**TGA**	STOP	1.1	14
**CGA**	Arg	6	31
**TGC**	Cys	6.1	12
**CGG**	Arg	6.9	22
**TGT**	Cys	7.3	19
**CAC**	His	7.9	8
**ACG**	Thr	8.2	15
**CCC**	Pro	9.8	21
**TCG**	Ser	9.9	16

**Table 6:** *Tropheryma whipplei* str. Twist 16S ribosomal RNA

Codon	Codes as	Usage frequency ‰	Count
**TAA**	STOP	0.9	8
**TAG**	STOP	1	3
**TGA**	STOP	1.1	5
**CGA**	Arg	6	5
**TGC**	Cys	6.1	8
**CGG**	Arg	6.9	15
**TGT**	Cys	7.3	3
**CAC**	His	7.9	6
**ACG**	Thr	8.2	7
**CCC**	Pro	9.8	6
**TCG**	Ser	9.9	10

### Codon measurement

**Fig. 5:**
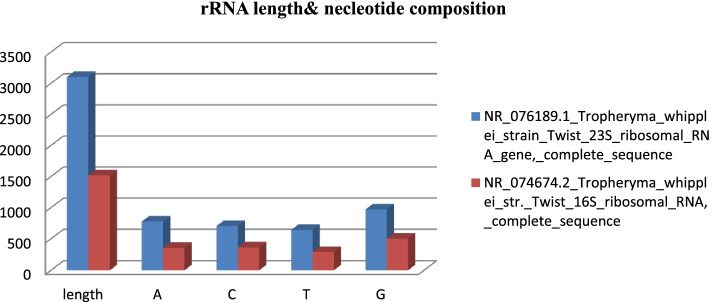
rRNA length and nucleotide composition

**Fig. 6:**
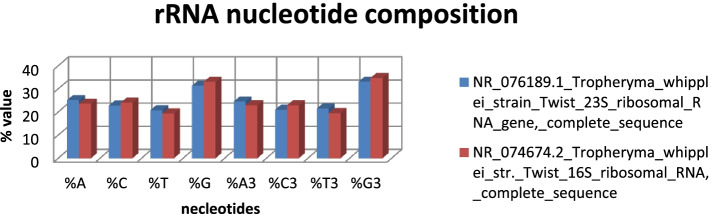
Percentage of rRNA nucleotide composition

**Fig. 7:**
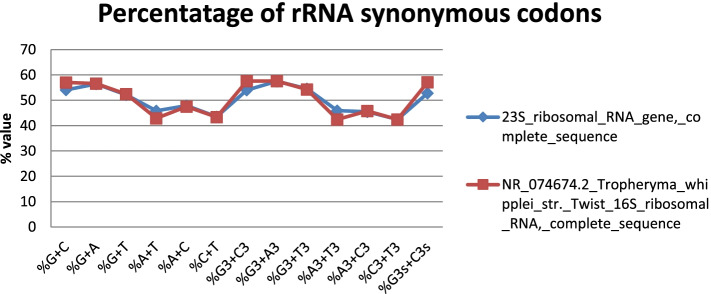
Percentage of rRNA synonymous codons

**Fig. 8:**
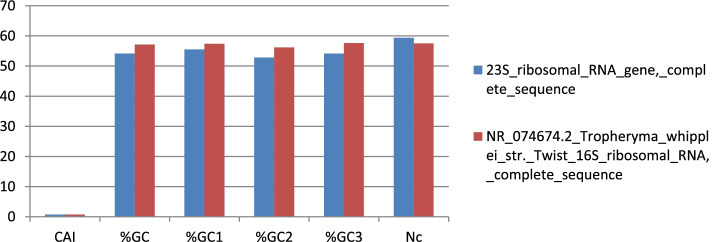
Codon measurement values

The calculated compositional properties for the coding sequences of the *Tropheryma whipplei* Twist strain are overall frequency of nucleotides A% (25.11 and 23.54), C% (22.76 and 24.0), T% (20.76 and 19.4), and G% (31.37 and 33.07) in 23s and 16s ribosomal RNA gene, respectively. The synonymous codons had the base content in 3rd position were calculated as A3S% (24.47 and 22.88), C3S% (20.99 and 22.88), T3S% (21.47 and 19.53), and G3S% (33.08 and 34.71) for 23s and 16s rRNA, respectively. GC3S% (52.85 and 57.85) is the third synonymous codon position in GC content of 23s and 16s rRNA, respectively. Figures 5 and 6 show rRNA characteristic features like length and nucleotide composition. In Fig. 7, rRNA synonymous codons percentage is given, while in Fig. 8, codon measurements were indicated.

### Epitope-based vaccine prediction: application of codon usage studies

**Fig. 9:**
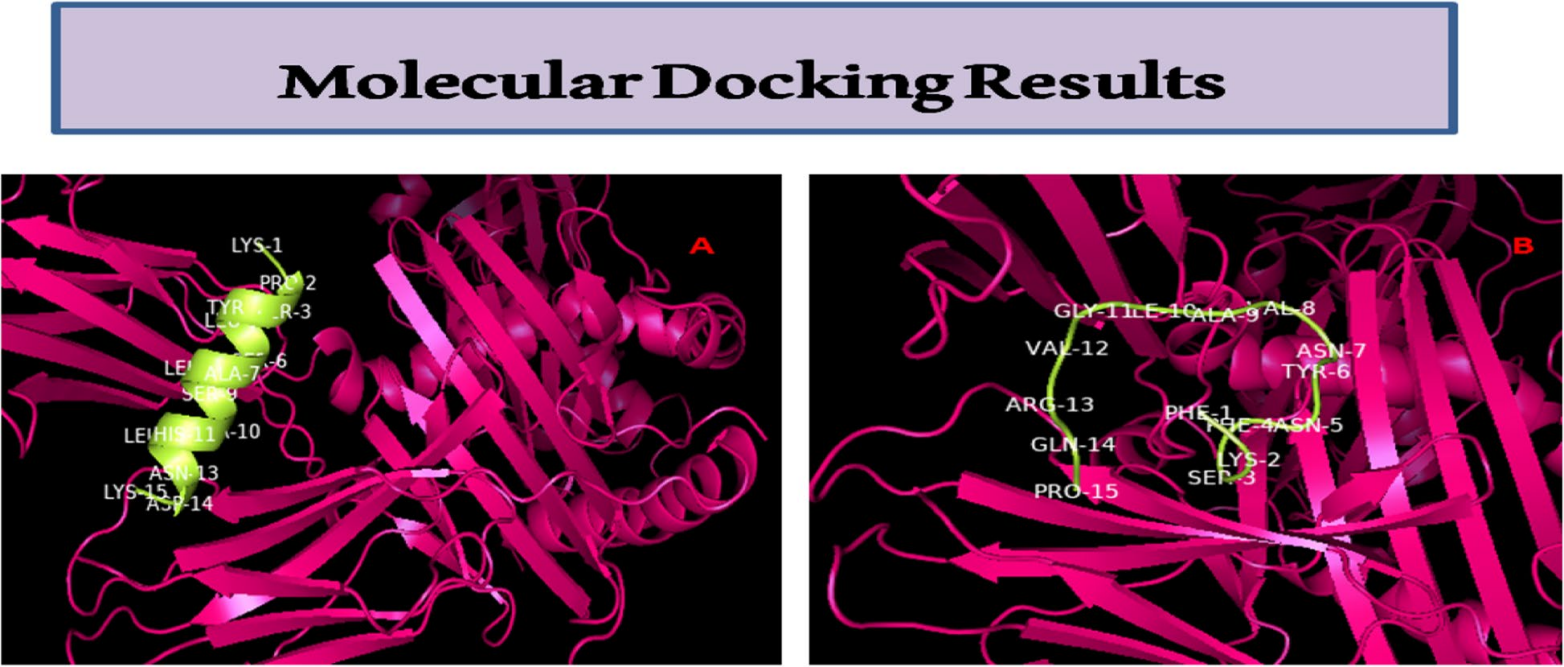
Molecular docking results of epitopes with HLA-DRB-0101. **A** KPSYLSALSAHLNDK from protein excinuclease ABC subunit UvrC and **B** FKSFNYNVAIGVRQP from protein 3-oxoacyl-ACP reductase FabG

The in silico analysis reveals two epitopes of 15 amino acid residues (i.e., KPSYLSALSAHLNDK and FKSFNYNVAIGVRQP) that hold perfect interaction with HLA-DRB-0101 (MHC class II allelic determinant). In Table 7, retrieved sequences were shown with accession numbers, and allergenicity was also presented by deploying Allergen FP tool (this tool generates Tanimoto similarity index). Epitopes were determined by using NetMHCIIpan-4.0 server that gathers core information from IEDB database and uses artificial neural networks (ANN) to access interaction of peptidal stretches to HLA allelic determinants. Amino acids like valine, aspartate, leucine, and phenylalanine hold high codon usage frequency and also found to be present in these screened epitopes from excinuclease ABC subunit UvrC and 3-oxoacyl-ACP reductase FabG. In Table 8, all 10 peptides are holding good VaxiJen score, and NetMHCIIpan-4.0 scores are provided, but there were a total of 2151 epitopes discovered. VaxiJen score indicates antigenicity for peptides. ProtParam results reveal only two finalized epitopes to be stable (Table 9). Epitopes structure was predicted by using PEP-FOLD 3.5 [20], and HLA allelic determinant HLA DRB1_0101 (PDB-ID:1AQD) was retrieved from RCSB-PDB database to perform molecular docking analysis. Molecular docking of selected epitopes with HLA-DRB0101 shows perfect interaction (Table 10). Figure 9 indicates docked complexes of selected epitopes with HLA-DRB-0101 visualized in PyMOL software.

**Table 7:** AllergenFP score and proteins considered for *Tropheryma whipplei*

Proteins/no. of amino acid residues	GenBank-accession no.	Function	Allergen FP score	Inference
Prolipoprotein diacylglyceryl transferase (*Tropheryma whipplei*) 272 aa protein	WP_042506957.1	Catalyzes the transition of the diacylglyceryl group from phosphatidylglycerol to the sulfhydryl group of the N-terminal cysteine of a prolipoprotein, the first step in the development of mature lipoproteins	0.87	Non-allergen
Excinuclease ABC subunit UvrC (*Tropheryma whipplei*) 607 aa protein	WP_042506954.1	DNA excision repair	0.82	Non-allergen
Holliday junction resolvase RuvX (*Tropheryma whipplei*) 145 aa protein	WP_042506082.1	Nuclease activity, rRNA processing	0.82	Non-allergen
Exodeoxyribonuclease VII large subunit (*Tropheryma whipplei*) 404 aa protein	WP_042506175.1	Degrades single-stranded DNA bidirectionally, first into massive acid-insoluble oligonucleotides, then into small acid-soluble oligonucleotides	0.82	Non-allergen
Isoprenyl transferase (*Tropheryma whipplei*) 249 aa protein	WP_042506056.1	Isopentenyl diphosphate (IPP) condensation with allylic pyrophosphates is catalyzed, resulting in a number of terpenoids.	0.80	Non-allergen
3-oxoacyl-ACP reductase FabG (*Tropheryma whipplei*) 238 aa protein	WP_011096407.1	Catalyzes the NADPH-dependent reduction of beta-ketoacyl-ACP substrates to beta-hydroxyacyl-ACP products, the first reductive step in the elongation cycle of fatty acid biosynthesis	0.82	Non-allergen
ABC transporter permease subunit (*Tropheryma whipplei*) 332 aa protein	WP_206536426.1	Transmembrane transportation of molecules	0.90	Non-allergen

**Table 8:** Peptides showing interaction to HLA-DRB0101, NETMHCII PAN 4.0 server results, and VaxiJen score

Pos	Peptide	ID	Score	Rank	VaxiJen score	Inference
39	NRRFIVLTGNREFTA	WP_042506957.1	0.958934	0.16	−0.4516	Nonantigenic
316	KPSYLSALSAHLNDK	WP_042506954.1	0.978324	0.06	0.7208	Antigenic
384	LQKYLNLNSLPVRIE	WP_042506954.1	0.968518	0.11	1.1646	Antigenic
580	IEDISALPGFGVKTA	WP_042506954.1	0.960251	0.15	0.7039	Antigenic
227	RDKIQAAQTVLSRSA	WP_042506954.1	0.805061	0.85	0.1459	Antigenic
77	EFSRFLVSSGVQVRF	WP_042506082.1	0.651559	1.60	0.4449	Antigenic
235	KTPLISAIGHEADRP	WP_042506175.1	0.966542	0.12	−0.0952	Nonantigenic
231	DDFWAALRAYSGRSR	WP_042506056.1	0.960550	0.15	0.2368	Antigenic
24	FKSFNYNVAIGVRQP	WP_011096407.1	0.916978	0.35	0.7126	Antigenic
3	PARFFFVSPLSCVKP	WP_206536426.1	0.691033	1.40	0.6685	Antigenic

**Table 9:** ProtParam results: biochemical properties of epitopes

Peptides	Molecular mass	pI	Gravy score	Aliphatic index	Instability index	Half life mammalian reticulocytes
KPSYLSALSAHLNDK	1643.86	8.51	−0.553	91.33	5.83	1.3 h
LQKYLNLNSLPVRIE	1800.13	8.59	−0.147	149.33	86.04	5.5 h
IEDISALPGFGVKTA	1517.74	4.37	0.573	110.67	62.39	20 h
FKSFNYNVAIGVRQP	1739.99	9.99	−0.180	71.33	24.99	1.1 h
PARFFFVSPLSCVKP	1695.06	9.57	0.673	71.33	61.23	> 20 h

**Table 10:** ACE VALUE, global energy, and binding energy for selected docked complexes (epitopes to HLA DRB0101)

Epitope	ACE value (Kcal/Mol)	Global energy (Kcal/Mol)	Binding energy (Kcal/Mol)
KPSYLSALSAHLNDK	−6.59	−36.93	−2.80
FKSFNYNVAIGVRQP	−3.79	−1.19	−3.40

## Discussion

The *Tropheryma whipplei* causes acute gastroenteritis to neuronal damages in *Homo sapiens*. Genomics and codon adaptation studies would be helpful advancements of disease evolution prediction, prevention, and treatment of disease. The codon-pair usage table and dinucleotide usage data were identified from the CoCoPUTs database [23, 24]. The ENC value computed in our analysis was 56.138, which means more than one codon was used for each amino acid. The ENC value should be ≤ 35 for significant codon bias [26]. *Tropheryma whipplei Twist* strain’s CDS, codons, frequency per thousand, and the number of codons; for identification of rare codons and sequence optimization. The ratio of observed codon frequency to the expected synonymous codons usage for the amino acid i.e., relative synonymous codon usage (RSCU) [28]. The degree of bias towards estimated, i.e., Codon Adaptation Index, value was 0.73 and 0.725 for 23s and 16s rRNA respectively. The value ranged between 0 and 1; higher values indicate stronger bias in codon usage and high gene expression level. In previous studies, membrane proteins were considered to be associated with considerable biasness [29], while in current study, we recognized rare codon biasness associated with entire genome of *T. whipplei*. The major requirement of codon biasness study assists in determining amino acids expressed patterns that can be linked to epitope-based vaccine predictions. In recent studies, for SARS-CoV2 [30, 31], dengue [32, 33], Nipah [34], Candida fungus [35], Canine circovirus [36], and Zika virus [37], vaccine predictions were found to be successful. So, codon usage pattern determination can be considered as the preliminary step before deploying any ANN (artificial neural networking)-based web server/tool like NetMHC server for screening essential epitopes of small peptidal length (8–12 amino acids). The calculated compositional properties for the coding sequences of the *Tropheryma whipplei* Twist strain overall frequency of nucleotides A% (25.11 23.54), C% (22.76 24.0), T % (20.76 19.4), and G% (31.37 and 33.07) in 23s and 16 s ribosomal RNA gene respectively. In silico analysis reveals two epitopes of 15 amino acid residues (i.e., KPSYLSALSAHLNDK and FKSFNYNVAIGVRQP) that hold perfect interaction with HLA-DRB-0101 (MHC class II allelic determinant); future scope holds linkers and adjuvants to be connected and solid-phase synthesis of these epitopes to further test these epitopes in model organisms. Recent developments in immunoinformatics show novel ways to predict epitope-based vaccine candidates and therapeutics against many harmful pathogens like *Candida auris* [35] and human cytomegalovirus [38]. Similarly, drug repurposing was made easy against harmful pathogens by deploying bioinformatic approaches [39]. Similarly, for animal models, viral pathogenic proteomes were screened for vaccine designing by deploying immunoinformatics [33, 36, 40]. This study is unique in terms of saving time and money for peptide-based vaccine crafting.

## Conclusions

Considerable biases in codon usage and amino acid usage indicate clearly that *T. whipplei* has a low codon bias. The synonymous codons had the base content in 3rd position were calculated as A3S% (24.47 and 22.88), C3S% (20.99 and 22.88), T3S% (21.47 and 19.53), and G3S% (33.08 and 34.71) for 23s and 16s rRNA, respectively. Also, codon-usage patterns clearly indicate that there will be less chances of variational or evolutionary alterations in *T. whipplei* genomic sets. The analysis could be targeted for disease evolution prediction, developing drugs, or vaccine candidates. We also found KPSYLSALSAHLNDK and FKSFNYNVAIGVRQP, two epitopes, can possibly act as vaccine candidates against *T. whipplei*. A future development requires wet-lab validations for these epitopes that are highly expressed in this bacterium and have therapeutic peptide formation capability.

## Data Availability

All data is provided in manuscript.

## Abbreviations

IEDB: Immune epitope database
CAI: Codon Adaptation Index
RNA: Ribonucleic acid
NCBI: *National Center for Biotechnology Information*
HLA: Human leuckocyte antigen
RSCU: Relative synonymous codon usage
MHC: Major histocompatibility complex

## References

[1] Raoult D, Ogata H, Audic S, Robert C, Suhre K, Drancourt M, Claverie JM (2003). Tropheryma whipplei Twist: a human pathogenic actinobacteria with a reduced genome. Genome Res.

[2] Keita AK, Raoult D, Fenollar F (2013). Tropheryma whipplei as a commensal bacterium. Future Microbiol.

[3] Dolmans RA, Boel CE, Lacle MM, Kusters JG (2017). Clinical manifestations, treatment, and diagnosis of Tropheryma whipplei infections. Clin Microbiol Rev.

[4] Moos V, Schmidt C, Geelhaar A, Kunkel D, Allers K, Schinnerling K, Ignatius R (2010). Impaired immune functions of monocytes and macrophages in Whipple’s disease. Gastroenterology.

[5] Lagier JC, Lepidi H, Raoult D, Fenollar F (2010). Systemic Tropheryma whipplei: clinical presentation of 142 patients with infections diagnosed or confirmed in a reference center. Medicine.

[6] Gorvel L, Al Moussawi K, Ghigo E, Capo C, Mege JL, Desnues B (2010). Tropheryma whipplei, the Whipple’s disease bacillus, induces macrophage apoptosis through the extrinsic pathway. Cell Death Dis.

[7] Bentley SD, Maiwald M, Murphy LD, Pallen MJ, Yeats CA, Dover LG (2003). Sequencing and analysis of the genome of the Whipple’s disease bacterium Tropheryma whipplei. Lancet.

[8] Lagier JC, Fenollar F, Lepidi H, Raoult D (2011). Evidence of lifetime susceptibility to Tropheryma whipplei in patients with Whipple’s disease. J Antimicrob Chemother.

[9] Fenollar F, Rolain JM, Alric L, Papo T, Chauveheid MP, van de Beek D, Raoult D (2009). Resistance to trimethoprim/sulfamethoxazole and Tropheryma whipplei. Int J Antimicrob Agents.

[10] Joshi A, Kaushik V (2021). In-silico proteomic exploratory quest: crafting T-cell epitope vaccine against Whipple’s disease. Int J Pept Res Ther.

[11] Zavala A, Naya H, Romero H, Musto H (2002). Trends in codon and amino acid usage in Thermotoga maritima. J Mol Evol.

[12] Lafay B, Atherton JC, Sharp PM (2000). Absence of translationally selected synonymous codon usage bias in Helicobacter pylori. Microbiology.

[13] Romero H, Zavala A, Musto H (2000). Codon usage in Chlamydia trachomatis is the result of strand-specific mutational biases and a complex pattern of selective forces. Nucleic Acids Res.

[14] Sharma P, Sharma P, Ahmad S, Kumar A (2022). Chikungunya virus vaccine development: through computational proteome exploration for finding of HLA and cTAP binding novel epitopes as vaccine candidates. Int J Pept Res Ther.

[15] Joshi A, Ray NM, Singh J, Upadhyay AK, Kaushik V (2022). T-cell epitope-based vaccine designing against Orthohantavirus: a causative agent of deadly cardio-pulmonary disease. Netw Model Anal Health Inform Bioinform.

[16] Daniel E, Onwukwe GU, Wierenga RK, Quaggin SE, Vainio SJ, Krause M (2015). ATGme: open-source web application for rare codon identification and custom DNA sequence optimization. BMC Bioinform.

[17] Dimitrov I, Naneva L, Doytchinova I, Bangov I (2014). AllergenFP: allergenicity prediction by descriptor fingerprints. Bioinformatics.

[18] Reynisson B, Alvarez B, Paul S, Peters B, Nielsen M (2020). NetMHCpan-4.1 and NetMHCIIpan-4.0: improved predictions of MHC antigen presentation by concurrent motif deconvolution and integration of MS MHC eluted ligand data. Nucleic Acids Res.

[19] Doytchinova IA, Flower DR (2007). VaxiJen: a server for prediction of protective antigens, tumour antigens and subunit vaccines. BMC Bioinform.

[20] Thévenet P, Shen Y, Maupetit J, Guyon F, Derreumaux P, Tuffery P (2012). PEP-FOLD: an updated de novo structure prediction server for both linear and disulfide bonded cyclic peptides. Nucleic Acids Res.

[21] Schneidman-Duhovny D, Inbar Y, Nussinov R, Wolfson HJ (2005). PatchDock and SymmDock: servers for rigid and symmetric docking. Nucleic Acids Res.

[22] Antunes DA, Moll M, Devaurs D, Jackson KR, Lizée G, Kavraki LE (2017). DINC 2.0: a new protein–peptide docking webserver using an incremental approach. Cancer Res.

[23] Alexaki A, Kames J, Holcomb DD, Athey J, Santana-Quintero LV, Lam PVN (2019). Codon and codon-pair usage tables (CoCoPUTs): facilitating genetic variation analyses and recombinant gene design. J Mol Biol.

[24] Athey J, Alexaki A, Osipova E, Rostovtsev A, Santana-Quintero LV, Katneni U (2017). A new and updated resource for codon usage tables. BMC Bioinform.

[25] Wright F (1990). The ‘effective number of codons’ used in a gene. Gene.

[26] Butt AM, Nasrullah I, Qamar R, Tong Y (2016). Evolution of codon usage in Zika virus genomes is host and vector specific. Emerg Microbes Infect.

[27] Seligmann H (2019). Localized context-dependent effects of the “ambush” hypothesis: more off-frame stop codons downstream of shifty codons. DNA Cell Biol.

[28] Sharp PM, Li WH (1986). Codon usage in regulatory genes in Escherichia coli does not reflect selection for ‘rare’codons. Nucleic Acids Res.

[29] Das S, Paul S, Dutta C (2006). Evolutionary constraints on codon and amino acid usage in two strains of human pathogenic actinobacteria Tropheryma whipplei. J Mol Evol.

[30] Joshi A, Joshi BC, Mannan MAU, Kaushik V (2020). Epitope based vaccine prediction for SARS-COV-2 by deploying immuno-informatics approach. Inform Med Unlocked.

[31] Akhtar N, Joshi A, Singh B, Kaushik V (2020) Immuno-informatics quest against COVID-19/SARS-COV-2: determining putative T-cell epitopes for vaccine prediction. Infect Disord Drug Targets. 10.2174/1871526520666200921154149 10.2174/1871526520666200921154149 32957905

[32] Krishnan S, Joshi A, Akhtar N, Kaushik V (2021). Immunoinformatics designed T cell multi epitope dengue peptide vaccine derived from non structural proteome. Microb Pathog.

[33] Krishnan S, Joshi A, Kaushik V (2020). T cell epitope designing for dengue peptide vaccine using docking and molecular simulation studies. Mol Simul.

[34] Kaushik V (2019) In silico identification of epitope-based peptide vaccine for Nipah virus. Int J Pept Res Ther 1-7. 10.1007/s10989-019-09917-0

[35] Akhtar N, Joshi A, Kaushik V, Kumar M, Mannan MAU (2021). In-silico design of a multivalent epitope-based vaccine against Candida auris. Microb Pathog.

[36] Jain P, Joshi A, Akhtar N, Krishnan S, Kaushik V (2021). An immunoinformatics study: designing multivalent T-cell epitope vaccine against canine circovirus. J Genet Eng Biotechnol.

[37] Sharma P, Kaur R, Upadhyay AK, Kaushik V (2020). In-silico prediction of peptide based vaccine against Zika virus. Int J Pept Res Ther.

[38] Akhtar N, Joshi A, Singh J, Kaushik V (2021) Design of a novel and potent multivalent epitope based human Cytomegalovirus peptide vaccine: an immunoinformatics approach. J Mol Liq 116586. 10.1016/j.molliq.2021.116586

[39] Joshi A, Krishnan GS, Kaushik V (2020). Molecular docking and simulation investigation: effect of beta-sesquiphellandrene with ionic integration on SARS-CoV2 and SFTS viruses. J Genet Eng Biotechnol.

[40] Joshi A, Pathak DC, Mannan MAU, Kaushik V (2021). In-silico designing of epitope-based vaccine against the seven banded grouper nervous necrosis virus affecting fish species. Netw Model Anal Health Inform Bioinform.

